# Epidemiology and outcomes associated with brain metastases among patients with metastatic breast cancer – a cohort study in US electronic health record data

**DOI:** 10.1186/s12885-025-14786-6

**Published:** 2025-10-01

**Authors:** Della Varghese, Jenna Collins, Beth Nordstrom, Miguel Miranda, Brian Murphy, David Harland

**Affiliations:** 1 Epidemiology, Vaccines & Immune Therapies, AstraZeneca, Gaithersburg, MD USA; 2 Evidera, Waltham, MA USA; 3 Statistical Sciences, AstraZeneca, Cambridge, UK; 4 Oncology Medical Affairs, AstraZeneca, Cambridge, UK

**Keywords:** Metastatic breast cancer, Brain metastases, Human epidermal growth factor receptor 2, HER2-positive, HER2-negative, Systemic therapy, Real world, Prevalence, Outcomes

## Abstract

**Background:**

There are limited real-world data on the prevalence of brain metastases (BM) in metastatic breast cancer (mBC) across the treatment pathway, especially when stratified by human epidermal growth factor receptor 2–positive (HER2+) or HER2–negative (HER2−) status. The goals of this study were to estimate the prevalence of BM at metastatic diagnosis and at the start of each line of systemic therapy (LOT), and to describe treatment patterns and overall survival (OS) in patients with and without BM.

**Methods:**

This retrospective cohort study included adult patients in the US with mBC diagnosed between January 2013 − May 2020, with known HER2 status from an electronic health record-derived, deidentified database. Patients were followed from mBC diagnosis to last activity date or death. Descriptive statistics were used for BM prevalence, patient characteristics, and treatment patterns. OS was estimated using the Kaplan-Meier method.

**Results:**

Of 12,644 patients with mBC in the database, 1923 (HER2+) and 9693 (HER2−) were included. The prevalence of BM at mBC diagnosis was 12.5% (HER2+) and 1.7% (HER2−). An NCCN Clinical Practice Guidelines in Oncology (NCCN Guidelines^®^) recommended systemic treatment for BM was received by 25.0% of patients with BM (HER2+) versus 12.8% (HER2−) during first-line treatment. The prevalence of BM (documented before or within the same month of LOT start) was 11.2%, 22.8%, and 33.0% in those with HER2+ diseases who had at least one, two, and three prior LOTs, respectively. The prevalence of BM among patients with HER2− disease was 1.6%, 2.0%, and 2.8% in those who had at least one, two, and three prior LOTs, respectively. Median OS from mBC diagnosis among patients with versus without BM was 24 versus 37 months (HER2+) and 12 versus 27 months (HER2−).

**Conclusions:**

In this real-world study of patients receiving care in US oncology clinics, the prevalence of BM in patients with mBC increased by LOT, and most were not receiving NCCN Guideline^®^-recommended systemic therapies. OS was poorer in patients with BM versus without BM, especially in the HER2− population. These results highlight a need for more effective treatments for patients with mBC and BM.

**Supplementary Information:**

The online version contains supplementary material available at 10.1186/s12885-025-14786-6.

## Background

Approximately 15−30% of patients with metastatic breast cancer (mBC) develop brain metastases (BM) [1, 2]. In patients who test positive for human epidermal growth factor receptor 2 (HER2) mBC, the incidence can be as high as 50% [3, 4]. Data for patients with HER2–negative (HER2−) mBC are more limited, but prevalence estimates range from 14 to 46% depending on hormone receptor (HR) status [5, 6]. BM are associated with an extremely poor prognosis and neurological deficits due to impaired cognitive and sensory functions in patients with mBC, which result in substantial morbidity and mortality [3, 4, 7, 8].

Local therapies form the backbone of treatment for BM, both in general [9], and in patients with mBC and BM [10, 11]. These can include surgical resection, whole-brain radiotherapy (WBRT), and stereotactic radiosurgery [9–11]. The local therapy used depends on the severity and extent of BM. Patients with few lesions may be eligible for surgery or stereotactic radiosurgery, but those with more extensive disease may require WBRT [10]. While WBRT may improve disease control, it does not improve overall survival (OS) and is associated with neurological decline [12–15]; hence, there is a need for systemic therapy options. Indeed, in spite of recent advances in both local and systemic therapies, outcomes for patients with BM and mBC did not improve between the year 2000 and 2020 [16].

Patients with mBC and BM may receive systemic therapies either in conjunction with local therapies or when local intervention is not indicated [10, 11]. Recommended treatments for patients with breast cancer (BC) and BM differ depending on HER2 status. At the present time, the combination of tucatinib, capecitabine, and trastuzumab is a recommended treatment option for patients with HER2–positive (HER2+) mBC and BM whose disease has progressed on at least one prior line of HER2-directed therapy [9, 10]. Other recommended therapies include trastuzumab deruxtecan (T-DXd), trastuzumab emtansine (T-DM1), capecitabine + lapatinib, capecitabine + neratinib, pertuzumab + trastuzumab, and paclitaxel + neratinib, but guidance on treatment sequencing is lacking [9, 10]. For patients with HER2− mBC and BM, recommended non-HER2-specific treatments include capecitabine, cisplatin, etoposide, cisplatin + etoposide, or high-dose methotrexate [9]. Although recommended treatments for patients with mBC are dependent on HR status [11], there is no guidance on whether HR status should play a role in treatment in the presence of BM [9, 11, 17]. Not all these therapies were available during the study described in this report; however, a list of the treatment options recommended in the 2021 NCCN Guidelines and how they were categorized is given in Table S1.

Prior research has reported the prevalence of BM at mBC diagnosis and corresponding median OS but has tended to focus on the HER2+ population [7, 18–21]. Further, limited contemporary data are available regarding the prevalence of BM by line of therapy (LOT) [21]. A better understanding of treatment patterns across the disease course may highlight any deviation from guideline practices, as well as identify areas where further guidance is needed. Data on ideal treatment sequencing are also lacking, and it is unclear, for example, whether effective treatments are being reserved for later LOTs in real-world clinical practice. The goals of this study were to estimate the prevalence of BM among patients with mBC at diagnosis and at the start of each LOT, by HER2 status, and to describe clinical outcomes for these patients. Treatment patterns at each LOT are also described, in addition to time to next treatment (TTNT) and time to discontinuation (TTD), which are pragmatic time-to-event endpoints for real-world data that may correlate with clinical benefit [22]. Recent developments in HER2-targeted therapies (tucatinib and T-DXd) mean it is desirable to gain a better understanding of disease course and treatment patterns in not only the HER2+ population, but also the HER2− population, which currently incorporates patients with HER2-low or HER2-ultralow disease who may be eligible for T-DXd [23].

## Methods

### Data source

The Flatiron Health database is an electronic health record-derived, deidentified database built from more than 265 cancer clinics broadly distributed across the US, as well as a limited number of National Comprehensive Cancer Network^®^ (NCCN^®^) member institutions. Flatiron Health electronic health record data are composed of processed longitudinal patient-level data (structured and unstructured) obtained from community and academic cancer clinics, providing information on patient demographics, diagnosis (e.g. staging, histopathology, and biomarkers), treatments, and outcomes (e.g. mortality) [24, 25].

### Study population

The full study cohort included patients aged ≥ 18 years who had histologically or cytologically documented BC with evidence of metastatic disease, with an initial metastatic diagnosis occurring between January 1, 2013, and May 31, 2020 (initial diagnosis of BC may have occurred earlier). The data cutoff was May 31, 2021, to allow for a minimum of 12 months of follow up. Patients were followed longitudinally until death or their last record of a visit prior to data cutoff (data censoring). Index date/baseline was defined as the date of initial mBC diagnosis. HER2 status was defined as confirmed HER2+ or HER2− status from immunohistochemistry (IHC) or fluorescence in situ hybridization (FISH). HER2+ was defined as any HER2+ test and the absence of a negative test (defined as IHC negative 0–1+, FISH negative/not amplified, negative not otherwise specified). HER2− was defined as any HER2− test and the absence of a positive test (IHC positive 3+, FISH positive/amplified, positive not otherwise specified). HR status was defined by biomarker results for either estrogen or progesterone receptor status at index and LOT start date. HR+ status was defined as positive estrogen and/or progesterone receptor status; HR− status was defined as negative status for both estrogen and progesterone receptors. Where HER2 status or HR status was not available, ‘unknown’ was reported. In cases of discrepant results, ‘other’ was reported.

### Baseline and treatment characteristics

Demographic and clinical characteristics, including tumor status and disease characteristics, were extracted from the electronic health records. Treatment patterns were described by collecting data on the type of BC systemic treatment (agent or combination of agents) received in the first four LOTs (1–4 L) and were categorized based on the NCCN Guidelines from 2021 [26, 27]. Treatments were categorized as BM-specific treatment (defined as NCCN Guideline-recommended regimens for the treatment of mBC with BM, though they can also be used for the treatment of mBC without BM) and non-BM specific (Table S1). Flatiron Health’s existing LOT algorithm, which is oncologist defined and rule based, was modified slightly to reflect current clinical practice for the patients with HER2+ mBC. Further details are reported in the supplementary information.

### Outcomes

#### Prevalence of BM

Month and year of BM diagnosis are noted in the Flatiron Health database. For patients with BM diagnosis in the same month as the mBC diagnosis, the date of mBC diagnosis was used as the date of BM diagnosis. For all other patients, the day of BM diagnosis was imputed as the 15th of the month. Prevalence of BM at diagnosis was defined as the number of patients with BM prior to or during the same month as the mBC diagnosis date, divided by the total number of patients. Patients were deemed to have BM at diagnosis if their date of BM was ≤ 30 days after their mBC diagnosis date. Similarly, prevalence of BM at the start of each LOT was calculated within each subgroup by taking the number of patients with BM prior to or during the same month as the start of the respective LOT, divided by the number of patients with at least that number of LOTs. Since incident cases of BM could not be definitively confirmed within the database, cumulative incidence was examined as an exploratory outcome and was reported at 12, 24, and 36 months. Patients were censored for the competing risk of death in the absence of a BM event; those without either event were censored at the last observed timepoint.

#### OS

OS for each patient was defined as the date of death minus the mBC diagnosis date or the start date of each LOT + 1. For patients with no record of death, OS was censored at the last activity date during the study period. Month and year of death are noted in the Flatiron Health database; the day of death was imputed as the maximum of the date of the mid-point of the month of death or the last activity date across all medical records in that month. The last activity date was considered as the latest date of a visit of any type (including for drug administration or laboratory test). Patients who had more than one instance of a record that occurred after the month of death had their survival censored as of the latest date of a visit of any type, as it was unclear whether the patient truly died during the month on record.

#### TTNT or death

TTNT was defined as the time from the start of the LOT to the start of the subsequent LOT or the date of death for patients who died without receiving a subsequent LOT. For patients with no indication of a further LOT or death, TTNT was censored at the last activity date during the study period.

#### TTD or death

TTD was defined as the time from the start of the LOT to the date of last administration + 21 days for injectable and infused therapies or end date of oral medication (identified from detailed oral files) or date of death + 1 for patients who died before the end of the LOT. For patients with no indication of discontinuation of the LOT or death, TTD was censored at the last visit date before the end of the study period if they were still on the medication at that point.

### Statistical analysis

All analyses were descriptive in nature. Categorical variables were reported using frequency counts and percentages for each category, while continuous variables were reported using mean, standard deviation, median, quartiles (Q1 and Q3), minimum, and maximum. The number of missing values was reported for each variable, and percentages for categorical variables were based on non-missing values. Kaplan-Meier methods were used for clinical outcome measures. Prevalence of BM at mBC diagnosis and at the start of each LOT was calculated. The 95% confidence intervals (CI) were calculated using the Clopper-Pearson method. Baseline characteristics, treatment patterns, and clinical outcomes were stratified for mBC patients with HER2+ and HER2− status.

## Results

### Baseline demographics and clinical characteristics

Of all individuals in the electronic health record database diagnosed with mBC between January 2013 and May 2020 (19,924), 12,644 patients with a HER2 biomarker test result and at least 1 day of follow up after index date were included. Within this population, 1923 patients with HER2+ mBC and 9693 patients with HER2− mBC were included in the study. Baseline characteristics and the proportion of patients with BM at the index date are presented in Table 1. The median age (range) at the index date was 61 (25–84) years in the overall HER2+ cohort and 64 (22–84) years in the overall HER2− cohort. Of all HER2+ patients with BM, 47.5% were Stage III at the initial BC diagnosis, with 30.0% at Stage II, and 58.3% had only one metastatic site at the first diagnosis of mBC. Among patients with BM in the HER2− cohort, 39.8% were Stage II and 38.0% were Stage III at initial BC diagnosis; almost half (47.6%) had one metastatic site, and 21.1% had two metastatic sites at initial mBC diagnosis (Table 1; Table S2).

**Table 1:** Baseline demographic and clinical characteristics by presence of BM at mBC diagnosis

	HER2+			HER2−		
	Overall *n* = 1923	Presence of BM at mBC diagnosis *n* = 240	Absence of BM at mBC diagnosis *n* = 1683	Overall *n* = 9693	Presence of BM at mBC diagnosis *n* = 166	Absence of BM at mBC diagnosis *n* = 9527
Age (years) at mBC diagnosis*						
Median (range)	61 (25–84)	55 (27–83)	62 (25–84)	64 (22–84)	60 (26–82)	64 (22–84)
**Sex**,** n (%)**						
Male	22 (1.1)	≤ 5 (NA)	21 (1.2)	109 (1.1)	0 (0.0)	109 (1.1)
Female	1901 (98.9)	≥ 235 (NA)	1662 (98.8)	9583 (98.9)	166 (100.0)	9417 (98.8)
**Race**,** n (%)**						
White	1248 (64.9)	166 (69.2)	1082 (64.3)	6435 (66.4)	104 (62.7)	6331 (66.5)
Black or African American	224 (11.6)	19 (7.9)	205 (12.2)	1137 (11.7)	27 (16.3)	1110 (11.7)
Asian	51 (2.7)	9 (3.8)	42 (2.5)	209 (2.2)	6 (3.6)	203 (2.1)
Other	256 (13.3)	29 (12.1)	227 (13.5)	1117 (11.5)	18 (10.8)	1099 (11.5)
Unknown	144 (7.5)	17 (7.1)	127 (7.5)	795 (8.2)	11 (6.6)	784 (8.2)
**Time (days) from initial BC diagnosis to mBC diagnosis**						
Number of observations	1922	240	1682	9683	166	9517
Mean (SD)	1473 (1536)	1105 (956)	1525 (1595)	1917 (1809)	1432 (1341)	1926 (1815)
Median (range)	999 (0–12,417)	812 (0–5889)	1028 (0–12,417)	1338 (0–14,893)	926 (0–6443)	1348 (0–14,893)
**Stage at initial BC diagnosis**,** n (%)**						
I	253 (13.2)	27 (11.3)	226 (13.4)	1581 (16.3)	27 (16.3)	1554 (16.3)
II	615 (32.0)	72 (30.0)	543 (32.3)	3763 (38.8)	66 (39.8)	3697 (38.8)
III	673 (35.0)	114 (47.5)	559 (33.2)	2946 (30.4)	63 (38.0)	2883 (30.3)
IV	159 (8.3)	6 (2.5)	153 (9.1)	480 (5.0)	5 (3.0)	475 (5.0)
Unknown	223 (11.6)	21 (8.8)	202 (12.0)	923 (9.5)	5 (3.0)	918 (9.6)
**Number of metastatic sites at any time prior to or on the date of mBC diagnosis**,** n (%)**						
1	961 (50.0)	140 (58.3)	821 (48.8)	1454 (15.0)	79 (47.6)	1375 (14.4)
2	400 (20.8)	37 (15.4)	363 (21.6)	663 (6.5)	35 (21.1)	598 (6.3)
3	226 (11.8)	30 (12.5)	196 (11.6)	264 (2.7)	24 (14.5)	240 (2.5)
4+	118 (6.1)	33 (13.8)	85 (5.1)	163 (1.7)	28 (16.9)	135 (1.4)
Unknown	218 (11.3)	0 (0.0)	218 (13.0)	7179 (74.1)	0 (0.0)	7179 (75.4)
**Sites of metastases at any time prior to or on the date of mBC diagnosis**,** n (%)**						
Bone	807 (42.0)	52 (21.7)	755 (44.9)	1480 (15.3)	55 (33.1)	1425 (15.0)
Brain	229 (11.9)	229 (95.4)	0 (0.0)	151 (1.6)	151 (91.0)	0 (0.0)
Liver	457 (23.8)	36 (15.0)	421 (25.0)	486 (5.0)	27 (16.3)	459 (4.8)
Lung	544 (28.3)	58 (24.2)	486 (28.9)	723 (7.5)	49 (29.5)	674 (7.1)
Other	754 (39.2)	62 (25.8)	692 (41.1)	1129 (11.6)	52 (31.1)	1077 (11.3)
Unknown	218 (11.3)	0 (0.0)	218 (13.0)	7179 (74.1)	0 (0.0)	7179 (75.4)
**HR status prior to or on the date of mBC diagnosis**,** n (%)**						
Positive	1290 (67.1)	134 (55.8)	1156 (68.7)	7564 (78.0)	90 (54.2)	7474 (78.5)
Negative	575 (29.9)	100 (41.7)	475 (28.2)	1951 (20.1)	75 (45.2)	1876 (19.7)
Other	7 (0.4)	2 (0.8)	5 (0.3)	35 (0.4)	0 (0.0)	35 (0.4)
Unknown	51 (2.7)	4 (1.7)	47 (2.8)	143 (1.5)	1 (0.6)	142 (1.5)

Cell entries show n (%), unless otherwise specified

*BC* Breast cancer, *BM* Brain metastases, *HER2−* Human epidermal growth factor receptor 2–negative, *HER2+* Human epidermal growth factor receptor 2–positive, *HR* Hormone receptor, *mBC* Metastatic breast cancer, *SD* Standard deviation

*Patients with a birth year of 1936 or earlier may have an adjusted birth year in Flatiron Health datasets due to patient deidentification requirements

### Prevalence of BM

The prevalence of BM at initial mBC diagnosis in the overall cohort of patients was 3.4%; at the start of 1 L, 2 L, and 3 L, prevalence of BM was 2.7%, 4.1%, and 5.3%, respectively (Table 2).

**Table 2:** Prevalence of BM, overall and stratified by HER2 status

Timepoint	Overall cohort	HER2+	HER2−
**mBC diagnosis**			
n in cohort	12,644	1923	9693
Prevalence of BM, % (95% CI)	3.4 (3.1–3.7)	12.5 (11.0–14.0)	1.7 (1.5–2.0)
**For HER2+, received treatment other than hormone therapy within 90 days after mBC diagnosis**			
n (%) patients	11,875 (93.9)	1154 (60.0)	NA
**Start of 1 L**			
n (%) with at least one LOT	10,546 (88.8)	1147 (99.4)	8516 (87.9)
Prevalence of BM, % (95% CI)	2.7 (2.4–3.0)	11.2 (9.4–13.1)	1.6 (1.3–1.9)
**Start of 2 L**			
n (%) with at least two LOTs	6799 (57.3)	646 (56.0)	5571 (57.5)
Prevalence of BM, % (95% CI)	4.1 (3.6–4.6)	22.8 (19.6–26.2)	2.0 (1.6–2.4)
**Start of 3 L**			
n (%) with at least three LOTs	4161 (35.0)	318 (27.6)	3474 (35.8)
Prevalence of BM, % (95% CI)	5.3 (4.6–6.0)	33.0 (27.9–38.5)	2.8 (2.2–3.4)

*1 L* First line, *2 L* Second line, *3 L* Third line, *BM* Brain metastases, *CI* Confidence interval, *HER2* Human epidermal growth factor receptor 2, *HER2−* Human epidermal growth factor receptor 2–negative, *HER2+* Human epidermal growth factor receptor 2–positive; *LOT* Line of therapy, *mBC* Metastatic breast cancer, *NA* Not applicable

#### HER2+ cohort

Among 1923 patients with HER2+ mBC, the prevalence of BM at the initial mBC diagnosis was 12.5%. Among 1147 patients with at least one LOT, 11.2% had BM at the start of 1 L treatment (Table 2). Prevalence of BM increased at later LOTs, being 22.8% at the start of 2 L and 33.0% at the start of 3 L.

#### HER2− cohort

At diagnosis, 1.7% of the 9693 patients included with HER2− mBC had BM; of these, 8516 received at least one LOT, and 1.6% had BM at the start of 1 L treatment. At the start of 2 L treatment, prevalence of BM was 2.0%; at the start of 3 L, it was 2.8% (Table 2). Prevalence of BM increased by LOT in both the HR+, HER2− and HR−, HER2− cohorts. In the HR+, HER2− group, 1.2% of 7564 patients had BM at index date, while 2.1% had BM at the start of 3 L treatment. For patients with HR−, HER2− mBC, the prevalence of BM at index was 3.8%, rising to 7.1% at the start of 3 L treatment (Table S3).

#### Cumulative incidence of BM

The cumulative incidence rate of BM (95% CI) among patients with HER2+ mBC who were BM free at mBC diagnosis was 0.081 (0.068–0.095) at 12 months, 0.161 (0.143–0.181) at 24 months, and 0.198 (0.178–0.219) at 36 months post-mBC diagnosis (Table S4; Figure S1). In the overall HER2− mBC cohort, cumulative incidence of BM was 0.011 (0.009–0.013) at 12 months, 0.022 (0.019–0.025) at 24 months, and 0.028 (0.024–0.032) at 36 months post-mBC diagnosis (Table S5).

### Treatment characteristics

#### HER2+ cohort

Among patients with HER2+ status who had BM at the time of mBC diagnosis, 98.4% had at least one LOT with systemic therapy, 50.0% had at least two LOTs, and 27.4% had at least three LOTs (Table 3). In patients without BM at the time of mBC diagnosis, 99.5% of patients had at least one LOT, with 56.7% and 27.6% having at least two and three LOTs, respectively. Among patients with BM at the start of 1 L treatment, 25.0% of patients received an NCCN Guideline-recommended BM-specific systemic regimen as 1 L treatment (Table 4); the most common systemic regimen was trastuzumab + pertuzumab + taxane (THP) (Table 5). Receipt of BM-specific treatment varied by LOT, being received by 65.3% of patients at 2 L, 47.6% of patients at 3 L, and 45.6% of patients at 4 L. The top three treatment regimens were broadly similar across patients with and without BM at all LOTs (Table 5); in 1 L therapy of HER2+ mBC, the most common regimens were THP, trastuzumab, and T-DM1. Corticosteroid use by LOT for each cohort is shown in Table S6.

**Table 3:** Number of treatment lines received by HER2 and BM status

	HER2+		HER2−	
	With BM *n* = 240	Without BM *n* = 1683	With BM *n* = 166	Without BM *n* = 9527
Total number of lines of systemic therapy, n (%)				
0	2 (1.6)	5 (0.5)	40 (24.1)	1137 (11.9)
1	60 (48.4)	441 (42.8)	59 (35.5)	2886 (30.3)
2	28 (22.6)	300 (29.1)	29 (17.5)	2068 (21.7)
3	19 (15.3)	154 (15.0)	15 (9.0)	1335 (14.0)
4	15 (12.1)	130 (12.6)	23 (13.9)	2101 (22.1)

*BM* Brain metastases, *HER2−* Human epidermal growth factor receptor 2–negative, *HER2+* Human epidermal growth factor receptor 2–positive

**Table 4:** Receipt of BM-specific systemic therapy* by HER2 status and LOT

	HER2+		HER2−	
	With BM	Without BM	With BM	Without BM
Proportion receiving BM-specific therapy, n/n receiving LOT (%)				
At 1 L	32/128 (25.0)	168/1019 (16.5)	17/133 (12.8)	762/8383 (9.1)
At 2 L	96/147 (65.3)	244/499 (48.9)	25/110 (22.7)	745/5461 (13.6)
At 3 L	50/105 (47.6)	88/213 (41.3)	32/96 (33.3)	584/3378 (17.3)
At 4 L	26/57 (45.6)	28/88 (31.8)	29/124 (23.4)	400/2000 (20.0)

*1 L* First line, *2 L* Second line, *3 L* Third line, *4 L* Fourth line, *BM* Brain metastases, *HER2−* Human epidermal growth factor receptor 2–negative, *HER2+* Human epidermal growth factor receptor 2–positive, *LOT* Line of therapy

*Based on the NCCN Guidelines for the treatment of BC and central nervous system cancers from 2021 (Table S1)

**Table 5:** Top regimens by LOT according to HER2 and BM status

	HER2+		HER2−	
	With BM	Without BM	With BM	Without BM
Top three regimens (%)				
At 1 L	THP (19.5) Trastuzumab (14.8) T-DM1 (10.9) *n* = 128	THP (27.2) T-DM1 (8.6) Trastuzumab (6.9) *n* = 1019	Anastrozole (11.3) Letrozole (7.5) Capecitabine (6.8) *n* = 133	Anastrozole (10.4) Letrozole + palbociclib (9.3) Letrozole (8.2) *n* = 8383
At 2 L	T-DM1 (33.3) Antimetabolite + lapatinib (9.5) THP (6.1) *n* = 147	T-DM1 (29.5) Hormone therapy (8.2) THP (7.8) *n* = 499	Capecitabine (8.2) Eribulin (6.4) Carboplatin + gemcitabine (6.4) *n* = 110	Fulvestrant + palbociclib (10.1) Capecitabine (8.3) Fulvestrant (7.8) *n* = 5461
At 3 L	T-DM1 (14.3) Antimetabolite + lapatinib (10.5) T-DXd (5.7) *n* = 105	T-DM1 (16.9) Antimetabolite + lapatinib (6.1) T-DXd (5.6) *n* = 213	Eribulin (9.4) Capecitabine (8.3) Fulvestrant (3.1) Paclitaxel (3.1) *n* = 96	Capecitabine (10.7) Fulvestrant + palbociclib (6.3) Eribulin (5.6) *n* = 3378
At 4 L+	T-DM1 (12.3) T-DXd (14.0) Antimetabolite + trastuzumab + tucatinib (14.0) *n* = 57	T-DM1 (11.4) T-DXd (9.1) Microtubule inhibitor (9.1) *n* = 88	Capecitabine (8.1) Eribulin (8.1) Gemcitabine (7.3) *n* = 124	Capecitabine (11.8) Eribulin (7.5) Paclitaxel protein bound (4.7) *n* = 2000

*n* = number of patients receiving LOT

*1 L* First line, *2 L* Second line, *3 L* Third line, *4 L +* Fourth line or later, *BM* Brain metastases, *HER2−* Human epidermal growth factor receptor 2–negative, *HER2+* Human epidermal growth factor receptor 2–positive, *LOT* Line of therapy, *T-DM1* Trastuzumab emtansine, *T-DXd* Trastuzumab deruxtecan, *THP* Trastuzumab + pertuzumab + taxane

#### HER2− cohort

Among patients with HER2− status who had BM at the time of mBC diagnosis, 75.9% received at least one LOT with systemic therapy, 40.4% received at least two LOTs, and 22.9% had at least three LOTs (Table 3). In patients without BM at the time of diagnosis, 88.1% had at least one LOT, with 57.8% and 36.1% having at least two and three LOTs, respectively. Among patients with BM at the start of 1 L treatment, 12.8% of patients were treated with an NCCN Guideline-recommended BM-specific therapy (Table 4). The proportion of patients with BM receiving a BM-specific treatment was higher at later lines, being 22.7% at 2 L, 33.3% at 3 L, and 23.4% at 4 L. At 1 L, anastrozole, letrozole, and capecitabine (in patients with BM) or letrozole + palbociclib (in patients without BM) were the most common treatment regimens (Table 5). Receipt of corticosteroids was higher in patients with BM versus without BM at all Lines of therapy, ranging from 42.9% at 1 L to 60.5% at 4 L (Table S6).

### Clinical outcomes

#### OS

##### HER2+ cohort

**Fig. 1:**
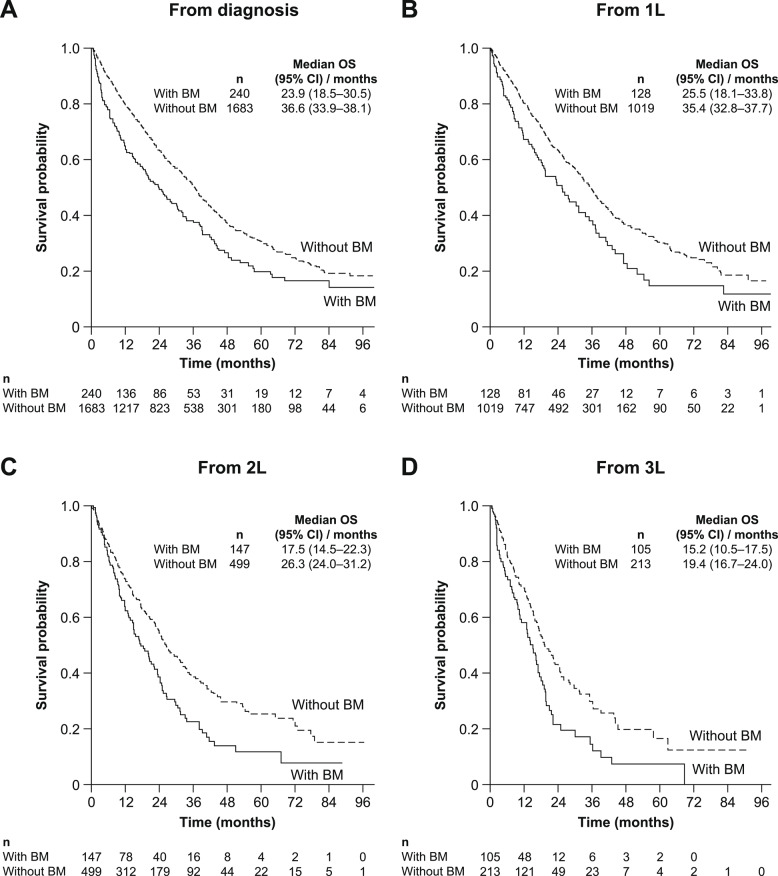
OS for HER2+ mBC populations with and without BM at diagnosis and by LOT (**A**) from mBC diagnosis date, (**B**) from start of 1 L treatment, (**C**) from start of 2 L treatment, (**D**) from start of 3 L treatment. *1 L* First line, *2 L* Second line, *3 L* Third line, *BM* Brain metastases, *CI* Confidence interval, *HER2+* Human epidermal growth factor receptor 2–positive, *LOT* Line of treatment, *mBC* Metastatic breast cancer, *OS* Overall survival

OS for the HER2+ cohort from mBC diagnosis and from the start of each LOT is presented in Fig. 1. Median OS (95% CI) from mBC diagnosis was 23.9 (18.5–30.5) months for patients with BM and 36.6 (33.9–38.1) months for patients without BM. Median OS (95% CI) from the start of 1 L therapy was 25.5 (18.1–33.8) months for patients with BM and 35.4 (32.8–37.7) months for patients without BM. From the start of 2 L therapy, the median OS (95% CI) was 17.5 (14.5–22.3) months for patients with BM; for the group without BM, it was 26.3 (24.0–31.2) months.

##### HER2− cohort

**Fig. 2:**
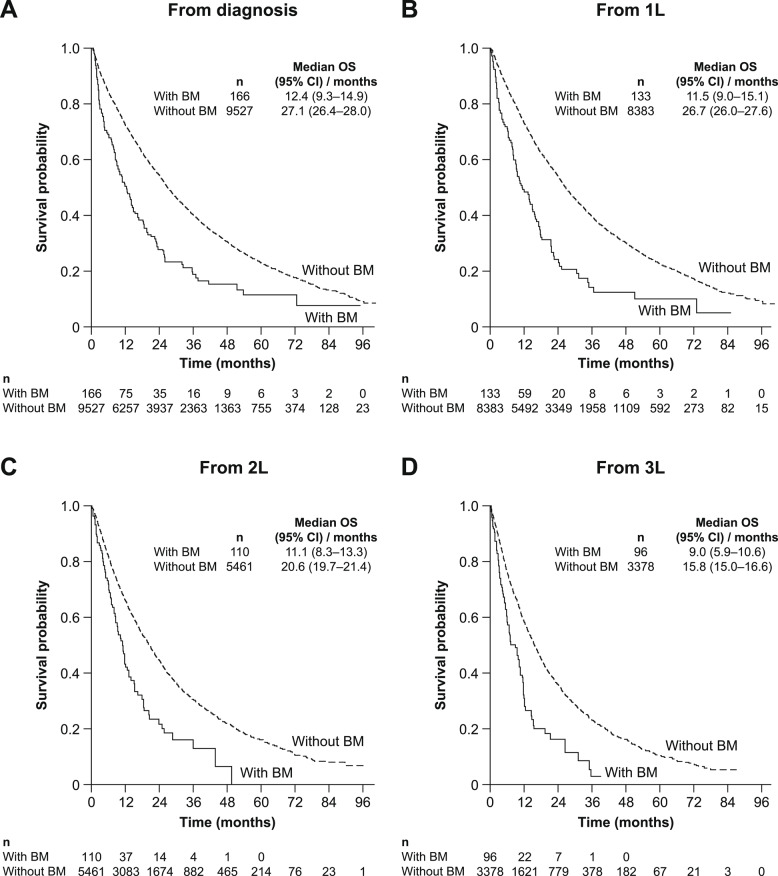
OS for the HER2− mBC population with and without BM at diagnosis and by LOT (**A**) from mBC diagnosis date, (**B**) from start of 1 L treatment, (**C**) from start of 2 L treatment, (**D**) from start of 3 L treatment. *1 L* First line, *2 L* Second line, *3 L* Third line, *BM* Brain metastases, *CI* Confidence interval, *HER2−* Human epidermal growth factor receptor 2–negative, *LOT* Line of treatment, *mBC* Metastatic breast cancer, *OS* Overall survival

OS for the HER2− cohort from mBC diagnosis and from the start of each LOT is presented in Fig. 2. Median OS (95% CI) from mBC diagnosis was 12.4 (9.3–14.9) months for patients with BM and 27.1 (26.4–28.0) months for patients without BM. At 1 L, median OS (95% CI) was 11.5 (9.0–15.1) months for patients with BM and 26.7 (26.0–27.6) months for patients without BM. From the start of 2 L therapy, the median OS (95% CI) was 11.1 (8.3–13.3) months for patients with BM; for the group without BM, it was 20.6 (19.7–21.4) months.

In the HR+, HER2− cohort, median OS (95% CI) from mBC diagnosis was 13.6 (8.9–18.7) months for patients with BM and 32.8 (31.8–33.8) months for patients without BM. In the HR−, HER2− cohort, patients with BM had a median OS (95% CI) from mBC diagnosis of 10.0 (8.0–14.9) months; for the group without BM, it was 12.0 (11.3–12.9) months.

#### TTNT

##### HER2+

**Fig. 3:**
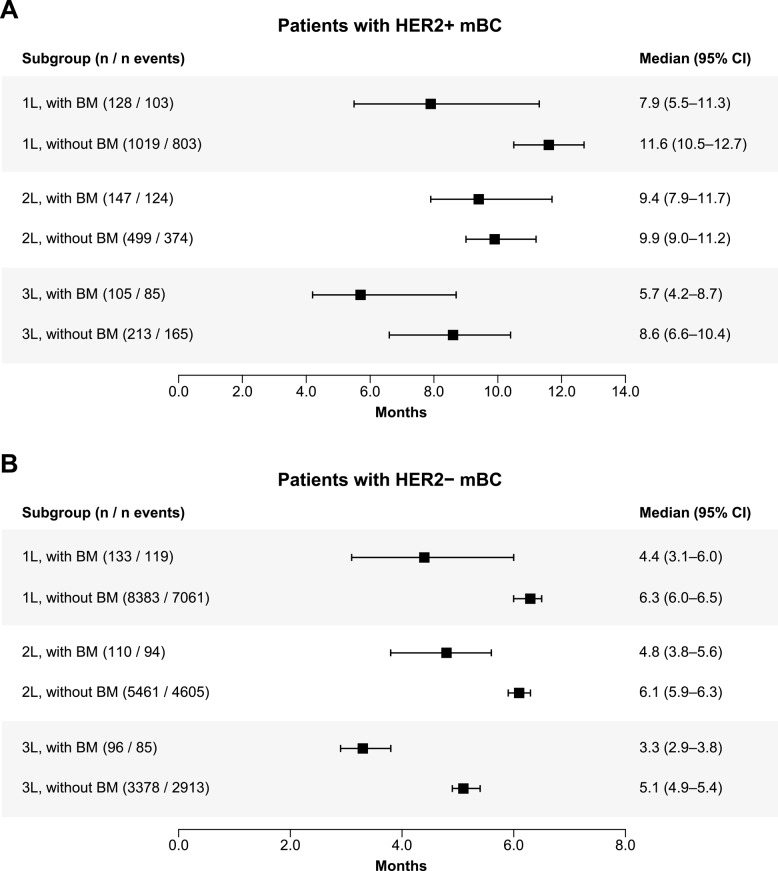
TTNT in patients with and without BM by LOT (**A**) HER2+ mBC population, (**B**) HER2 − mBC population. *1 L* First line, *2 L* Second line, *3 L* Third line, *BM* Brain metastases, *CI* Confidence interval, *HER2−* Human epidermal growth factor receptor 2–negative, *HER2+* Human epidermal growth factor receptor 2–positive, *LOT* Line of treatment, *mBC* Metastatic breast cancer, *TTNT* Time to next treatment

The median TTNT (95% CI) from the start of 1 L therapy was 7.9 (5.5–11.3) months for patients with BM and 11.6 (10.5–12.7) months for those without. For patients with BM, the median TTNT (95% CI) was 9.4 (7.9–11.7) and 5.7 (4.2–8.7) months for 2 L and 3 L, respectively. For patients without BM, the median TTNT (95% CI) was 9.9 (9.0–11.2) and 8.6 (6.6–10.4) months for 2 L and 3 L, respectively (Fig. 3).

##### HER2−

The median TTNT (95% CI) from the start of 1 L therapy was 4.4 (3.1–6.0) months for patients with BM and 6.3 (6.0–6.5) months for those without. For patients with BM, the median TTNT (95% CI) was 4.8 (3.8–5.6) and 3.3 (2.9–3.8) months for 2 L and 3 L, respectively. For patients without BM, the median TTNT (95% CI) was 6.1 (5.9–6.3) and 5.1 (4.9–5.4) months for 2 L and 3 L, respectively (Fig. 3).

#### TTD

##### HER2+

**Fig. 4:**
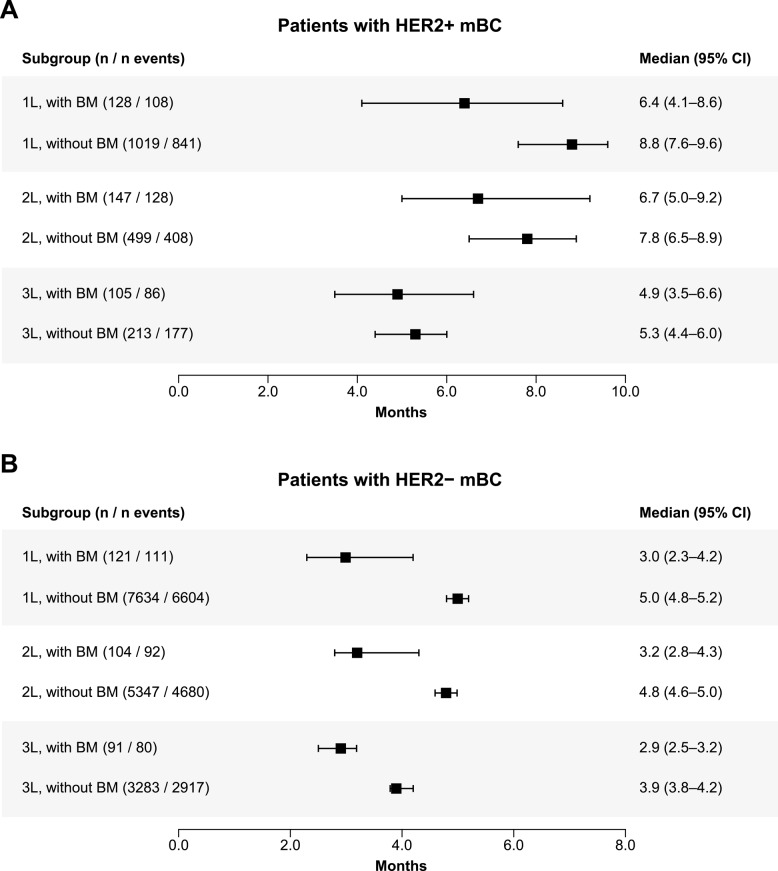
TTD in patients with and without BM by LOT (**A**) HER2+ mBC population, (**B**) HER2 − mBC population. *1 L* First line, *2 L* Second line, *3 L* Third line, *BM* Brain metastases, *CI* Confidence interval, *HER2−* Human epidermal growth factor receptor 2–negative, *HER2+* Human epidermal growth factor receptor 2–positive, *LOT* Line of treatment, *mBC* Metastatic breast cancer, *TTD* Time to treatment discontinuation

In patients with BM, the median TTD (95% CI) ranged from 6.4 (4.1–8.6) months from the start of 1 L therapy to 6.7 (5.0–9.2) months at 2 L and 4.9 (3.5–6.6) months at 3 L. The median TTD (95% CI) for patients without BM was 8.8 (7.6–9.6) months from the start of 1 L, 7.8 (6.5–8.9) months from the start of 2 L, and 5.3 (4.4–6.0) months from the start of 3 L (Fig. 4).

##### HER2−

The median TTD (95% CI) for patients with BM ranged from 3.0 (2.3–4.2) months from the start of 1 L therapy to 3.2 (2.8–4.3) months at 2 L and 2.9 (2.5–3.2) months at 3 L. For patients without BM, the median TTD (95% CI) was 5.0 (4.8–5.2) months from the start of 1 L, 4.8 (4.6–5.0) months at 2 L, and 3.9 (3.8–4.2) months at 3 L (Fig. 4).

## Discussion

This retrospective study examined a cohort of patients with mBC under routine clinical practice using an electronic health record-derived deidentified database. The prevalence of BM at first mBC diagnosis was 3.4% for the overall population. In both the HER2+ and HER2− populations, the prevalence of BM appeared to increase by LOT. However, the effect was more pronounced in the HER2+ cohort, ranging from 12.5% at mBC diagnosis (any point prior to 30 days after diagnosis) and 11.2% at the start of 1 L to 33.0% at the start of 3 L. The prevalence of BM was lower among patients with HER2− mBC, ranging from 1.7% at the time of mBC diagnosis and 1.6% at the start of 1 L to 2.8% at the start of 3 L. The apparent increase in prevalence of BM by LOT could be a result of the ‘survival effect’, whereby patients who progress onto later lines of treatment are likely to have been living with cancer for longer than those who did not, and therefore, have spent more time at risk of developing brain metastases. Further, it is possible that patients who progress onto subsequent lines of therapy are more likely to be screened for BM, or otherwise have their BM detected, than those earlier in their disease course. However, there may also be other factors which could contribute to this observation.

In a recent study using the same database, including patients diagnosed with HER2+ mBC from January 2016 to May 2019, DeBusk et al. found a prevalence of BM at mBC diagnosis of 9.9% [7]. However, this study included BM present at the mBC diagnosis date. In contrast, the current study considered patients as having BM at mBC diagnosis if their date of BM was within 30 days after their diagnosis date to account for gaps in data capture. A database study of US community oncology practices which included adult, female patients diagnosed with mBC between 2012 and 2018 found 22% of patients to have BM at the time of mBC diagnosis, with a 39% prevalence during 1 L treatment and a 14% prevalence during 2 L treatment. The difference in prevalences found by this study compared with other reports and results reported here may be because the population included only patients who received at least two lines of systemic therapy, and therefore may not be representative of the overall HER2+ population. It should also be noted that the Vidal study included only 372 patients, compared with 1923 patients with HER2+ mBC in this study [21]. Other national studies have reported that 12–15% of patients with HER2+ mBC have BM at the time of mBC diagnosis [18, 19]. Prevalence data for the overall HER2− population have not been widely reported, but the prevalences for HR+, HER2− and HR−, HER2− subgroups in the current study are considerably lower than were reported in a study using the Surveillance, Epidemiology, and End Results (SEER) database of the National Cancer Institute from 2010 to 2013, which included patients with HR+, HER2− (5.5%) and HR−, HER2− (11.4%) breast cancer [20]. Of note, the prevalence given in the SEER study is for patients with BM at diagnosis of de novo mBC, where most patients in our study had mBC that had progressed from an earlier stage. In addition to providing more recent data, our study addresses gaps in the SEER study by reporting on patients with recurrent disease and describing BM prevalence across the treatment pathway [20].

In general, survival was poorer for patients with BM than those without, and also poorer for patients with HER2− disease than for patients with HER2+ disease. Further, our study results also seem to indicate that patients with BM generally had reduced OS versus patients without BM, independent of the timing of the BM diagnosis or treatments received. Median OS was 23.9 months for patients with HER2+ mBC and BM at the diagnosis date and 36.6 months for patients without BM at mBC diagnosis. These results are similar to those from the Debusk study [7], although median OS by LOT was shorter than that reported in the Vidal study, where a median was not reached in all groups except patients with BM at diagnosis in 1 L (53.0 months) [21]. In patients with HER2− mBC, survival was also poorer in patients with BM than in those without, being 12.4 versus 27.1 months, respectively. The median OS in patients with HR+, HER2− mBC and BM is consistent with data from the SEER database (13.6 vs. 14.0 months), but was found to be longer in this study for the HR−, HER2− group (10.0 vs. 6.0 months) [20]. The poor outcomes for patients with HER2− disease as a whole highlight the unmet need in this population, despite the low overall prevalence of BM in this group.

Both TTNT and TTD were longer for patients without BM than for patients with BM in both HER2+ and HER2− mBC. The heterogeneity of the patient population, censoring of patients who did not receive a next treatment or who had no indication of discontinuation or death, and variability of treatment in real-world clinical practice compared with carefully controlled clinical trials mean that these time-to-event outcomes may not be expected to align with those expected based on the literature. The TTNT at 1 L in patients with HER2+ mBC and BM (7.9 months) was consistent with the median progression-free survival (mPFS) reported in the 2014–2020 cohort from a retrospective observational study [16]. However, the TTNT in patients with HR+, HER2− mBC (6.9 months in 1 L treatment of patients without BM) was somewhat shorter than mPFS (> 10 months) reported in clinical studies of letrozole and anastrozole in 1 L [28–32].

Regarding treatment patterns for patients in this analysis, three-quarters of patients with HER2+ mBC and BM at the start of 1 L were not receiving NCCN Guideline-recommended BM-specific systemic therapies. Use of these recommended therapies was higher beyond 1 L therapy, with 65.3%, 47.6%, and 45.6% receiving them as 2 L, 3 L, and 4 L + therapy, respectively. DeBusk et al. found that only 11.8% of patients with HER2+ mBC and BM were receiving NCCN Guideline-recommended treatments in 1 L, increasing to 13.0% at 2 L, 16.9% at 3 L, and then decreasing to 9% at 4 L [7]. While the low use of recommended therapies appears consistent across the US community practices that contribute data to the electronic health record-derived deidentified database, the smaller uptake of recommended systemic therapies reported in the Debusk et al. analysis may be attributed to a narrower definition of BM-recommended treatment than was used in our study [7].

In the population with HER2− mBC, the majority of patients did not receive an NCCN Guideline-recommended systemic treatment at any LOT. At 1 L, 87% did not receive a recommended therapy. The proportion receiving a recommended therapy was slightly higher in later lines, being around one-quarter in 2 L and 4 L+, and around one-third in 3 L. At all LOTs, the proportion of patients with BM receiving an NCCN Guideline-recommended therapy for BM was numerically lower for patients with HER2− disease than for HER2+. Results indicate a general prioritization of treatment to the primary tumor over BM-specific therapies, and our data suggest a more specific unmet need in the HER2− patient population. However, these results must be interpreted cautiously owing to the lack of data on radiographical or surgical treatments. Indeed, data from the SEER database between 2014 and 2016 found that among patients > 65 years of age, around half received non-stereotactic brain-directed radiation, ~15% received stereotactic brain-directed radiation, and 10–20% received surgical resection [33]. In the same study, fewer than one-fifth of patients received systemic therapy in the absence of local therapy [33]. Similarly, a US single-center cohort study reported that between 2013 and 2015, only 10% of patients received no radiation therapy and, separately, that 27% received surgery [34].

The most common regimens received among patients with HER2+ mBC were broadly aligned with the Debusk et al. and Vidal et al. analyses [7, 21], as well as with treatment guidelines in place at the time of the study (i.e. before the approval of T-DXd for patients with HER2+ mBC) that suggested use of THP in 1 L and T-DM1 in 2 L [35]. Consistent with previous reports, trastuzumab-based therapies were commonly used across all lines of therapy, which may suggest that alternative treatment options are limited [7, 21].

In the HER2− population, the most common 1 L regimens were anastrozole and letrozole, regardless of BM status. In 2 L, fulvestrant + palbociclib, capecitabine monotherapy, and fulvestrant monotherapy were most common in the overall population and in the group without BM at the start of the line. Those with BM received capecitabine monotherapy and eribulin monotherapy most often at 2 L, which is somewhat consistent with the 2021 NCCN Guidelines that suggest capecitabine monotherapy, cisplatin, or etoposide (either alone or in combination) or high-dose methotrexate for non-HER2-targeted treatment of BM [26]. As in the HER2+ population, the same treatments were used across most LOTs in the groups with and without BM (capecitabine, fulvestrant, and eribulin), which may indicate a lack of treatment options, or a lack of guidance on optimal treatment sequencing. Nonetheless, the more commonly used treatments are reflective of those recommended in general according to HR status, with aromatase inhibitors, fulvestrant, and standard chemotherapy being effective in the HR+, HER2− population, while those who are HR−, HER2− may benefit from capecitabine, eribulin, or carboplatin plus bevacizumab [36].

The large cohorts constructed in the analyses reported here allowed for reasonable precision estimates. Additionally, few other databases exist with the equivalent timeliness of data availability. The results of the study are generalizable to a wide range of outpatient oncology practice groups but may not represent all sites in the US and are subject to limitations. The most significant of these is the focus on systemic therapy and the lack of information on surgery or radiation therapy, which remain the standard of care for patients with BM [10, 11]. Further, other services could have been underreported or missing, and treatments, services, and procedures provided outside of the Flatiron Health network may not be captured.

The HER2+ results include patients who had HER2+ disease as of their mBC diagnosis and do not account for any subsequent change in HER2 status. Another limitation of the study design is that we were unable to make direct comparisons between subgroups; multivariate analyses would have been informative to control for potential confounding variables and hence make any conclusions regarding survival differences more robust. A comparison between outcomes (for example, OS) in both HER2+ and HER2− groups, and between HR+, HER2− and HR−, HER2−, would be a valuable future addition to the literature. Recent developments in the treatment and diagnosis of mBC are also unaccounted for, including the subcategorization of HER2 expression levels in patients previously classified as HER2−.

Prevalence of BM diagnosis also needs to be interpreted with caution. Without per-protocol or frequent brain scans, prevalence of BM may be underestimated by those who received and/or reported scans; it is possible that a higher incidence would be found if all patients received routine screening at baseline and prior to initiation of a new LOT. Prevalence of BM by LOT may also have been affected by variable rates of screening or BM detection at each LOT. The true prevalence cannot be determined because any patients without a known HER2 status were excluded from the overall analysis. Rates of testing for BM were not available in the data.

Overall, this study provides a comprehensive examination of the prevalence of BM, treatment patterns, and other clinical outcomes of patients with mBC, stratified by HER2 status. The results contribute information on the frequency of BM across the treatment pathway, as other literature on this is scarce.

## Conclusion

This real-world study of patients receiving care in US oncology clinics reports the prevalence of BM in patients with HER2+ and HER2− mBC, both at diagnosis and by LOT. Prevalence of BM increased by LOT in both the HER2+ and HER2− cohorts. OS was poorer in patients with BM than in patients without BM and was also found to be poorer in patients with HER2− mBC and BM than patients with HER2+ mBC and BM. Over the course of this study, most mBC patients with BM were not receiving NCCN Guideline-recommended systemic treatments at 1 L. Receipt of NCCN Guideline-recommended treatment for BM varied by LOT; the proportion of patients receiving a recommended therapy was lower in the HER2− group than the HER2+ group at every LOT. This fact, combined with the worse outcomes seen for patients with HER2− mBC, suggests a specific unmet need in this patient population, while the generally poorer survival for patients with BM than those without, regardless of receipt of an NCCN Guideline-recommended regimen, highlights the need for more effective treatments.

## Supplementary Information

Supplementary Material 1.

## Data Availability

The data that support the findings of this study were originated by and are the property of Flatiron Health, Inc., which has restrictions prohibiting the authors from making the dataset publicly available. Requests for data sharing by license or by permission for the specific purpose of replicating results in this manuscript can be submitted to PublicationsDataAccess@flatiron.com. The data were deidentified and subject to obligations to prevent reidentification and protect patient confidentiality.

## Abbreviations

1L: First line
2L: Second line
3L: Third line
4L: Fourth line
BC: Breast cancer
BM: Brain metastases
CI: Confidence interval
FISH: Fluorescence in situ hybridization
HER2: Human epidermal growth factor receptor 2
HER2−: Human epidermal growth factor receptor 2–negative
HER2+: Human epidermal growth factor receptor 2–positive
HR: Hormone receptor
IHC: Immunohistochemistry
LOT: Line of therapy
mBC: Metastatic breast cancer
NCCN: National Comprehensive Cancer Network
OS: Overall survival
SEER: Surveillance, Epidemiology, and End Results
T-DM1: Trastuzumab emtansine
T-DXd: Trastuzumab deruxtecan
THP: Trastuzumab + pertuzumab + taxane
TTD: Time to discontinuation
TTNT: Time to next treatment
WBRT: Whole-brain radiotherapy
