# Supplementary material for: Epidemiology and outcomes associated with brain metastases among patients with metastatic breast cancer – a cohort study in US electronic health record data
Source: BMC Cancer. 2025 Oct 1;25:1475. doi: 10.1186/s12885-025-14786-6 (PMC12486881; doi:10.1186/s12885-025-14786-6)
Supplement: Supplementary file 1 — Supplementary Material 1. [file 12885_2025_14786_MOESM1_ESM.docx]

**SUPPLEMENTARY MATERIAL**

[METHODS 2](#_Toc194420403)

[Baseline and treatment characteristics 2](#_Toc194420404)

[SUPPLEMENTARY TABLES 3](#_Toc194420405)

[Table S1. Categorization of BC treatments in this study 3](#_Toc194420406)

[Table S2. Baseline demographics and clinical characteristics in patients with HR+, HER2− and HR−, HER2− mBC 4](#_Toc194420407)

[Table S3. Prevalence of BM stratified by HR status in patients with HER2− mBC 6](#_Toc194420408)

[Table S4. Cumulative incidence of BM among patients with HER2+ mBC who were BM free at mBC diagnosis 7](#_Toc194420409)

[Table S5. Cumulative incidence of BM among patients with HER2− mBC who were BM free at mBC diagnosis 8](#_Toc194420410)

[Table S6. Patients receiving corticosteroids by LOT 9](#_Toc194420411)

[SUPPLEMENTARY FIGURES 10](#_Toc194420412)

[Figure S1. Cumulative incidence of BM with death as a competing risk 10](#_Toc194420413)

# METHODS

## Baseline and treatment characteristics

Flatiron Health’s existing LOT algorithm, which is oncologist defined and rule based, was modified slightly to reflect current clinical practice for the patients with HER2+ mBC. Gaps in exposure of up to a year were allowed within the same LOT, with the following constituting the start of a new LOT: the addition of a new HER2-targeted drug or discontinuation of all HER2-targeted drugs, changes in chemotherapy or immunotherapy subclass, or the start of a new poly-adenosine diphosphate-ribose polymerase inhibitor or a cyclin-dependent kinase 4/6 inhibitor, combined with hormonal therapy. All other changes to a regimen, including the addition of hormonal therapy alone, did not constitute a new LOT. Patients with HER2+ mBC were also required to have received treatment other than hormone therapy within 90 days of mBC diagnosis, since patients with a 1L initiation later than clinically expected may be a reflection of 1L treatment outside the Flatiron Health network. For injectable and infused therapies, the medication administration file was used to derive the end date, which was the date of last administration + 21 days (the most common duration between administered treatments). The detailed oral file was used to identify the end date of oral medication. The end date of multidrug LOTs was the latest of these dates for the component agents.

# SUPPLEMENTARY TABLES

## **Table S1**. Categorization of BC treatments in this study

| **BM specific*** | | **Non-BM specific^†^** |
| --- | --- | --- |
| **HER2+ specific** | **HER2 non-specific** |  |
| Capecitabine + lapatinib | Capecitabine | Poly-adenosine diphosphate-ribose polymerase-containing regimen |
| Capecitabine + neratinib | Cisplatin | Immuno-oncology-containing regimen |
| Paclitaxel + neratinib | Etoposide | Cyclin-dependent kinase 4/6-containing regimen |
| Tucatinib + trastuzumab + capecitabine | Cisplatin + etoposide | Other HER2-targeted regimens |
| T-DM1 | High-dose methotrexate | Other chemotherapy regimens |
|  | Temozolomide^‡^ | Hormonal therapy without chemotherapy or HER2-targeted regimens |
|  |  | Other |

*BM* Brain metastases; *HER2* Human epidermal growth factor receptor 2; *HER2+* Human epidermal growth factor receptor 2–positive; *NCCN* National Comprehensive Cancer Network; *T-DM1* Trastuzumab emtansine

*Referenced with permission from the NCCN Clinical Practice Guidelines in Oncology (NCCN Guidelines^®^) for Central Nervous System Cancers, V.2.2021. © National Comprehensive Cancer Network, Inc 2021. All rights reserved. Accessed 2021. To view the most recent and complete version of the guideline, go online to NCCN.org

^†^Referenced with permission from the NCCN Clinical Practice Guidelines in Oncology (NCCN Guidelines^®^) for Breast Cancer, V.5.2021. © National Comprehensive Cancer Network, Inc 2021. All rights reserved. Accessed 2021. To view the most recent and complete version of the guideline, go online to NCCN.org

^‡^Temozolomide is listed as a tumor-agnostic therapy in the NCCN Guidelines for Central Nervous System Cancers

NCCN makes no warranties of any kind whatsoever regarding their content, use or application and disclaims any responsibility for their application or use in any way

## **Table S2**. Baseline demographics and clinical characteristics in patients with HR+, HER2− and HR−, HER2− mBC

|  | **HR+, HER2−** | | | **HR−, HER2−** | | |
| --- | --- | --- | --- | --- | --- | --- |
|  | **Overall*  n=7564** | **Presence of BM at mBC diagnosis n=90** | **Absence of BM at mBC diagnosis n=7474** | **Overall*  n=1951** | **Presence of BM at mBC diagnosis n=75** | **Absence of BM at mBC diagnosis n=1876** |
| **Age (years) at mBC diagnosis^†^** | | | | | | |
| Median (range) | 65  (23–84) | 63  (26–81) | 65  (23–84) | 61  (22–84) | 56 (34–82) | 61  (22–84) |
| **Sex,** **n (%)** |  |  |  |  |  |  |
| Male | 105  (1.4) | 0  (0.0) | 105  (1.4) | ≤5 (NA) | 0  (0.0) | ≤5 (NA) |
| Female | 7458 (98.6) | 90  (100.0) | 7368 (98.6) | ≥1946 (99.8) | 75 (100.0) | ≥1871  (99.8) |
| **Race, n (%)** |  |  |  |  |  |  |
| White | 5184 (68.5) | 59  (65.6) | 5125 (68.6) | 1135 (58.2) | 44 (58.7) | 1091  (58.2) |
| Black or African American | 708 (9.4) | 10 (11.1) | 698 (9.3) | 404 (20.7) | 17 (22.7) | 387 (20.6) |
| Asian | 162  (2.1) | 4  (4.4) | 158  (2.1) | 46 (2.4) | 2 (2.7) | 44 (2.3) |
| Other | 881 (11.6) | 10 (11.1) | 871 (11.7) | 218 (11.2) | 8 (10.7) | 210 (11.2) |
| Unknown | 629  (8.3) | 7  (7.8) | 622  (8.3) | 148 (7.6) | 4 (5.3) | 144 (7.7) |
| **Time (days) from initial BC diagnosis to mBC diagnosis** | | | | | | |
| Number of observations | 7555 | 90 | 7465 | 1950 | 75 | 1875 |
| Mean (SD) | 2142  (1855) | 1910 (1553) | 2145 (1859) | 1077 (1191) | 870 (707) | 1085 (1206) |
| Median (range) | 1642 (0–14893) | 1412 (0–6443) | 1642 (0–14893) | 698 (0–9943) | 639 (0–3963) | 699 (0–9943) |
| **Stage at initial BC diagnosis, n (%)** | | | | | | |
| I | 1271 (16.8) | 12 (13.3) | 1259 (16.8) | 292 (15.0) | 14 (18.7) | 278 (14.8) |
| II | 2981 (39.4) | 39 (43.3) | 2942 (39.4) | 742 (38.0) | 27 (36.0) | 715 (38.1) |
| III | 2230 (29.5) | 34 (37.8) | 2196 (29.4) | 679 (34.8) | 29 (38.7) | 650 (34.6) |
| IV | 354 (4.7) | 3 (3.3) | 351 (4.7) | 63 (3.2) | 2 (2.7) | 61 (3.3) |
| Unknown | 728 (9.6) | 2 (2.2) | 726 (9.7) | 175 (9.0) | 3 (4.0) | 172 (9.2) |
| **Number of metastatic sites at any time prior to or on the date of mBC diagnosis, n (%)** | | | | | | |
| 1 | 1143  (15.1) | 38 (42.2) | 1105 (14.8) | 291  (14.9) | 41 (54.7) | 250 (13.3) |
| 2 | 514 (6.8) | 25 (27.8) | 489 (6.5) | 109 (5.6) | 10 (13.3) | 99 (5.3) |
| 3 | 199 (2.6) | 11 (12.2) | 188 (2.5) | 60 (3.1) | 13 (17.3) | 47 (2.5) |
| 4+ | 122 (1.6) | 16 (17.8) | 106 (1.4) | 38 (1.9) | 11 (14.7) | 27 (1.4) |
| Unknown | 5586 (73.8) | 0 (0.0) | 5586 (74.7) | 1453 (74.5) | 0  (0.0) | 1453 (77.5) |
| **Sites of metastases at any time prior to or on the date of mBC diagnosis, n (%)** | | | | | | |
| Bone | 1286 (17.0) | 34 (37.8) | 1252 (16.8) | 172 (8.8) | 20 (26.7) | 152 (8.1) |
| Brain | 81 (1.1) | 81 (90.0) | 0  (0.0) | 69 (3.5) | 69 (92.0) | 0  (0.0) |
| Liver | 384 (5.1) | 18 (20.0) | 366 (4.9) | 92 (4.7) | 8 (10.7) | 84 (4.5) |
| Lung | 502 (6.6) | 25 (27.8) | 477  (6.4) | 212 (10.9) | 23 (30.7) | 189 (10.1) |
| Other | 850 (11.2) | 28 (31.1) | 822 (11.0) | 260 (13.3) | 24 (32.0) | 236 (12.6) |
| Unknown | 5586 (73.8) | 0  (0.0) | 5586 (74.7) | 1453 (74.5) | 0  (0.0) | 1453 (77.5) |

*BC* Breast cancer; *BM* Brain metastases; *HER2−* Human epidermal growth factor receptor 2–negative; *HER2+* Human epidermal growth factor receptor 2–positive; *HR* Hormone receptor; *mBC* Metastatic breast cancer; *NA* Not applicable; *SD* standard deviation

*Excludes patients with ‘unknown’ or ‘other’ HR status

^†^Patients with a birth year of 1936 or earlier may have an adjusted birth year in Flatiron Health datasets due to patient deidentification requirements

## **Table S3.** Prevalence of BM stratified by HR status in patients with HER2− mBC

| **Timepoint** | **HR+, HER2−** | **HR−, HER2−** |
| --- | --- | --- |
| **mBC diagnosis** |  |  |
| n in cohort | 7564 | 1951 |
| Prevalence of BM, % (95% CI) | 1.2  (1.0–1.5) | 3.8 (3.0–4.8) |
| n (%) patients | NA | NA |
| **Start of 1L** |  |  |
| n (%) with at least one LOT | 6910 (91.4) | 1148 (74.2) |
| Prevalence of BM, % (95% CI) | 1.2 (1.0–1.5) | 3.2 (2.4–4.3) |
| **Start of 2L** |  |  |
| n (%) with at least two LOTs | 4623 (61.1) | 842 (43.2) |
| Prevalence of BM, % (95% CI) | 1.5 (1.2–1.9) | 4.6 (3.3–6.3) |
| **Start of 3L** |  |  |
| n (%) with at least three LOTs | 2944 (38.9) | 466 (23.9) |
| Prevalence of BM, % (95% CI) | 2.1 (1.7–2.7) | 7.1 (4.9–9.8) |

*1L* First line; *2L* Second line; *3L* Third line; *BM* Brain metastases; *CI* Confidence interval; *HER2−* Human epidermal growth factor receptor 2–negative; *HER2+* Human epidermal growth factor receptor 2–positive; *HR* Hormone receptor; *LOT* Line of therapy;
*mBC* Metastatic brain cancer; *NA* Not applicable

## **Table S4.** Cumulative incidence of BM among patients with HER2+ mBC who were BM free at mBC diagnosis

| **Timepoint** | **Number of patients with  the outcome** | **Total number of  person-years** | **Cumulative incidence rate (95% CI)** |
| --- | --- | --- | --- |
| 12 months | 129 | 1389 | 0.081 (0.068–0.095) |
| 24 months | 244 | 2302 | 0.161 (0.143–0.181) |
| 36 months | 288 | 2868 | 0.198 (0.178–0.219) |

*BM* Brain metastases; *CI* Confidence interval; *HER2+* Human epidermal growth factor receptor 2–positive; *mBC* Metastatic breast cancer

## **Table S5**. Cumulative incidence of BM among patients with HER2− mBC who were BM free at mBC diagnosis

| **Timepoint** | **Number of patients with  the outcome** | **Total number of  person-years** | **Cumulative incidence rate (95% CI)** |
| --- | --- | --- | --- |
| 12 months | 98 | 7658 | 0.011 (0.009–0.013) |
| 24 months | 183 | 12612 | 0.022 (0.019–0.025) |
| 36 months | 225 | 15632 | 0.028 (0.024–0.032) |

*BM* Brain metastases; *CI* Confidence interval; *HER2−* Human epidermal growth factor receptor 2–negative; *mBC* Metastatic breast cancer

## **Table S6**. Patients receiving corticosteroids by LOT

|  | **HER2+** | | **HER2−** | |
| --- | --- | --- | --- | --- |
| **LOT** | **With BM n / N (%)** | **Without BM n / N (%)** | **With BM**  **n / N (%)** | **Without BM**  **n / N (%)** |
| 1 | 63 / 128  (49.2) | 634 / 1019 (62.2) | 57 / 133  (42.9) | 2093 / 8383 (25.0) |
| 2 | 66 / 147  (44.9) | 221 / 499  (44.3) | 53 / 110  (48.2) | 1622 / 5461 (29.7) |
| 3 | 56 / 105  (53.3) | 97 / 213  (45.5) | 42 / 96  (43.8) | 1191 / 3378 (35.3) |
| 4 | 32 / 57  (56.1) | 50 / 88  (56.8) | 75 / 124  (60.5) | 775 / 2000  (38.8) |

*BM* Brain metastases; *HER2−* Human epidermal growth factor receptor 2–negative;
*HER2+* Human epidermal growth factor receptor 2–positive; *LOT* Line of treatment

# SUPPLEMENTARY FIGURES

## **Figure S1**. Cumulative incidence of BM with death as a competing risk

A) patients with HER2+ mBC and B) patients with HER2− mBC


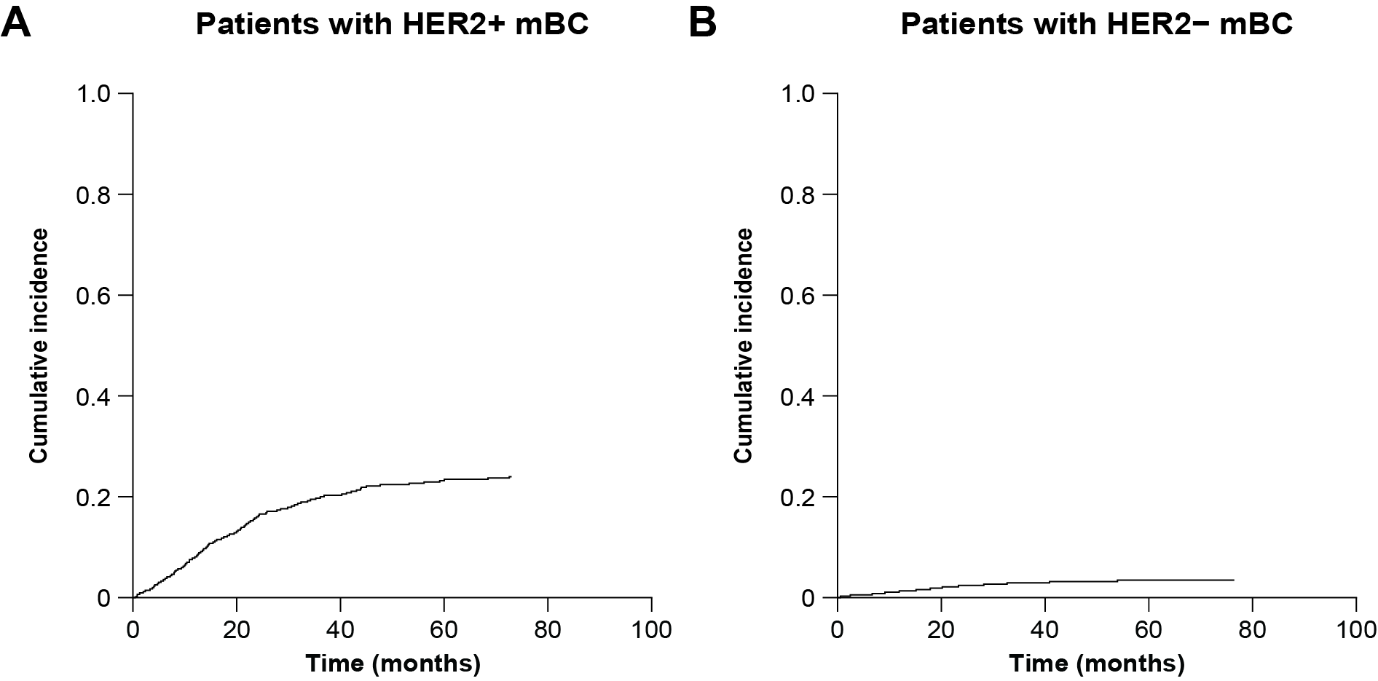


*BM* Brain metastases; *HER2−* Human epidermal growth factor receptor 2–negative; *HER2+* Human epidermal growth factor receptor 2–positive; *mBC* Metastatic breast cancer
